# Dataset of UV induced changes in nuclear proteome obtained by GeLC-Orbitrap/MS in *Pinus radiata* needles

**DOI:** 10.1016/j.dib.2016.03.074

**Published:** 2016-04-07

**Authors:** Sara Alegre, Jesús Pascual, Matthias Nagler, Wolfram Weckwerth, María Jesús Cañal, Luis Valledor

**Affiliations:** a Plant Physiology Lab, Organisms and Systems Biology, Faculty of Biology, University of Oviedo, Oviedo, Asturias, Spain; b Department of Ecogenomics and Systems Biology, Faculty of Life Sciences, University of Vienna, Vienna, Austria; c Vienna Metabolomics Center (VIME), University of Vienna, Vienna, Austria

## Abstract

Although responses to UV stress have been characterised at system and cellular levels, the dynamics of the nuclear proteome triggered in this situation are still unknown, despite its essential role in regulating gene expression and in last term plant physiology. To fill this gap, we characterised the variations in the nuclear proteome after 2 h and 16 h (8 h/day) of UV irradiation by using state-of-the-art mass spectrometry-based shotgun proteomics methods combined with novel bioinformatics workflows that were employed in the manuscript entitled “The variations in the nuclear proteome reveal new transcription factors and mechanisms involved in UV stress response in *Pinus radiata*” (Pascual et al., 2016) [1]. We employed in-gel digestion followed by a 120 min gradient prior to MS analysis. Data was processed following two approaches: a database dependent employing the SEQUEST algorithm and custom databases, and a database independent by mass accuracy precursor alignment (MAPA). 388 proteins were identified by SEQUEST search and 9094 m/z were quantified by MAPA. Significant m/z were *de novo* sequenced using the Novor algorithm. We present here the complete datasets and the analysis workflow.

**Specifications Table**

**Table:** 

Subject area	*Biology*
More specific subject area	*Plant Proteomics/Plant Biochemistry/Plant Stress response*
Type of data	*Tables and figures*
How data was acquired	*GE-nLC-LTQ-Orbitrap XL/MS*
Data format	*Filtered*
Experimental factors	*Pine trees were acclimated to grow under artificial light for* 3 *weeks in a greenhouse completely blinded to natural light. We wanted to avoid any UV irradiation coming from the sun.*
Experimental features	*Pinus radiata plants were treated with UV light* (0.33 W m^−2^). *Two different treatments were applied:* 8 h/*day of irradiation during two consecutive days, and* 2 h *of irradiation. The two experiments finished at the same time to avoid potential circadian interferences. Proteins were extracted, in-gel trypsin digested, and resolved in an Orbitrap XL employing a* 120 min *gradient.*
Data source location	*Oviedo, Principality of Asturias, Spain*
Data accessibility	*Data is available within this article.*

**Value of the data**

• First report of the nuclear proteome of *Pinus radiata.*
• Combination of database dependent and independent approaches to increase proteome coverage.
• A new workflow for analysing difficult samples of non-sequenced species is provided.
• A new set of UV stress-responsive proteins were discovered.

## 1 Data

The dataset provided consists of pre-processed tables belonging to the analysis of nuclear-enriched fractions from UV-treated *Pinus radiata* needles performed in “The variations in the nuclear proteome reveal new transcription factors and mechanisms involved in UV stress response in *Pinus radiata*” (Pascual et al., 2016) [1]. Three data points (control, 2 h of irradiation, and 16 h of irradiation at 8 h/day) and three biological replicates were analysed. We provide a quantification table based on SEQUEST protein identification and the quantification of the three most abundant peaks of each protein (default quantification method in Proteome Discoverer 1.4). In this table, identification, coverage, and quantification information are included (Supplemental Table 1). The information in this table has been pre-processed and only nuclear proteins are included.

The second table (Supplemental Table 2) shows the normalised abundance of all m/z parent masses considered in this work after a Mass Accuracy Precursor Alignment (MAPA) analysis and pre-processing.

## 2 Experimental design, materials and methods

### 2.1 Plant material and experimental design

The assay was conducted on one-year-old seedlings. After cold stratification, seeds were germinated and grown in peat:perlite:vermiculite (3:1:1) in 1.5 L pots in a greenhouse under a long photoperiod (16 h light/8 h darkness). Plants were irradiated with 0.33 W m^−2^ UV light (250–350 nm fluorescent tube, peaking at 300 nm) in independent blocks of 3 individuals. Samples were taken at different intervals: after 2 h of irradiation (2h samples) and after 16 h of irradiation during two consecutive days (8 h each day, 16h samples). Control plants were maintained under the same greenhouse conditions without UV radiation (Control). To avoid biases related to circadian clock, all the plants were sampled at the same time. Plant material was sampled, immediately frozen in liquid nitrogen and stored at −80 °C until analysis.

### 2.2 Nuclei isolation and protein extraction

Nuclei were isolated following the protocol described by Valledor et al. [2] with minor modifications. In brief, 1 g of needles was ground to powder using a mortar and liquid nitrogen, immediately extracted with 10 mL of buffer A (0.44 M sucrose, 10 mM Tris–HCl pH 8.0, 5 mM β-mercaptoethanol and 0.015 mM PMSF), filtered, centrifuged at 3,000*g* and the supernatant was discarded. Then, the samples were incubated in buffer B (0.25 M sucrose, 10 mM Tris–HCl pH 8.0, 10 mM MgCl_2_, 1% Triton X-100, 5 mM β-mercaptoethanol and 0.015 mM PMSF), centrifuged at 3,000*g* and the supernatant was discarded. This step was repeated until whitish pellets were obtained (if pellets were greenish after three washes we increased the Triton concentration). Afterwards, samples were washed with buffer C (0.25 M sucrose, 10 mM Tris–HCl pH 8.0, 10 mM MgCl_2_, 5 mM β-mercaptoethanol and 0.015 mM PMSF) and centrifuged to remove supernatant. Finally, pellets were dissolved carefully in 400 µL of buffer C:ddH_2_O (2:1, v/v) and cleared by centrifugation in a discontinuous sucrose gradient: 0.32 M sucrose, 3 mM CaCl_2_, 2 mM Mg(C_2_H_3_O_2_)_2_, 0.1 mM EDTA, 10 mM Tris–HCl pH 8, 1 mM DTT, 0.5% (v/v) NP-40; 2 M sucrose, 5 mM Mg(C_2_H_3_O_2_)_2_, 0.1 mM EDTA, 10 mM Tris–HCl pH 8, 1 mM DTT; and 3 M sucrose, 5 mM Mg(C_2_H_3_O_2_)_2_, 0.1 mM EDTA, 10 mM Tris–HCl pH 8, 1 mM DTT; autoclaved and NP-40 and DTT added just prior to use. Thereafter, the samples were centrifuged 12 min at 3000*g*. All the steps were performed at 4°C.

**Fig. 1:**
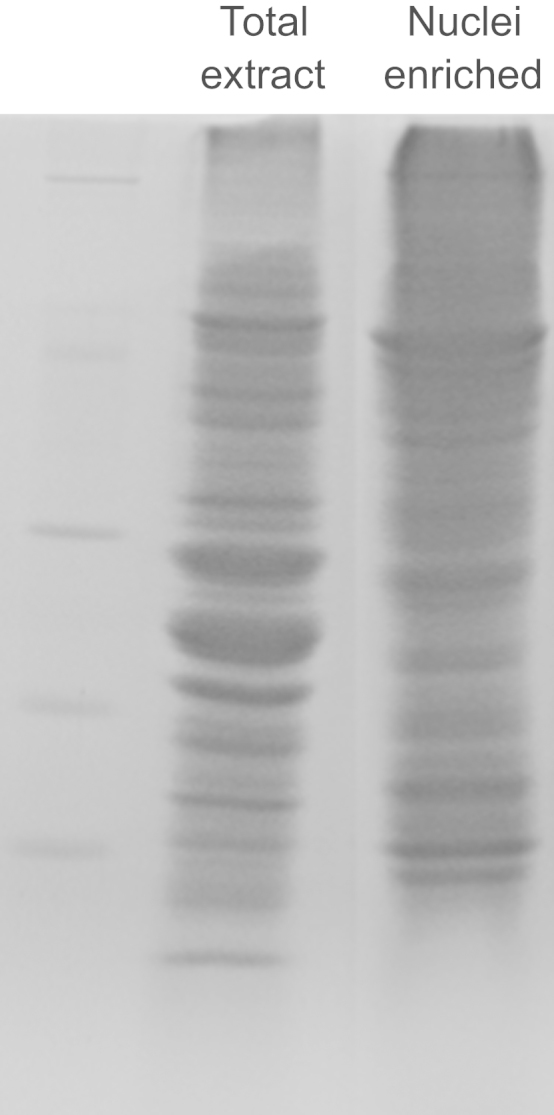
Comparison of 1-D SDS-PAGE profiles of total and nuclear-enriched protein fractions.

Once the nuclei were isolated, samples were sonicated in 300 μL of 1% SDS for 15 s at 60% amplitude (Hielcher UP200S) and then incubated in a vortex at maximum speed for 15 min at room temperature. Subsequently, one volume of buffer Z 1.5 M sucrose, 10 mM DTT and 1% protease inhibitor cocktail (Sigma, P9599) and one volume of phenol were added to each sample to prevent protein degradation. After mixing vigorously, tubes were centrifuged 5 min at 17,000*g* and room temperature. After centrifugation, phenolic (upper) phase was saved and the lower phase was re-extracted by adding one volume of phenol. Both phenolic phases were collected and cleaned with buffer Z in the same way to conserve only the upper phase. Proteins were precipitated by adding 2 v of 0.1 M ammonium acetate in methanol and incubated overnight at −20 °C. Tubes were centrifuged, and protein pellets washed twice with acetone. Dry pellets were dissolved in 1.5% SDS, 8 M Urea. Protein content was quantified by BCA assay [3]. The enrichment in nuclear proteins was assessed by 1-DE SDS-PAGE (Fig. 1).

### 2.3 Protein digestion and mass spectrometry

Proteins were processed and analysed as described by Valledor et al. [4] with minor modifications. Protein samples (60 µg) were run 0.5 cm in 12% SDS-PAGE gels. Gels were fixed and stained with 0.1% CBB R-250 in methanol:acetic acid:water (40:10:50, v/v/v) for 30 min and destained in methanol:water (40:60, v/v). Excised bands were chopped and washed twice in 1 mL of 50 mM ammonium bicarbonate:acetonitrile (2:1), once time in 1:1 proportion and finally dried out in 300 µL of acetonitrile. All washing steps were performed for 15 min in a shaker.

Once cleaned and dried, 75 µL of trypsin [12.5 ng/µL of trypsin in 25 mM NH_4_HCO_3_, 10% (v/v) acetonitrile, 5 mM CaCl_2_] were added to each sample. Hydrated gel pieces were covered with 25 mM NH_4_HCO_3_ (AmBic), 10% (v/v) acetonitrile, 5 mM CaCl_2_ and samples were incubated at 37 °C for 14 h. Gel pieces were washed twice with 250 µL of 25 mM ammonium bicarbonate, 50% acetonitrile, 0.1% formic acid. After each wash, the washing buffer was collected to recover the peptides. All fractions corresponding to each sample were placed in the same tube and dried in a speedvac. Peptides were then dissolved in 4% (vv) acetonitrile, 0.25% (v/v) formic acid and desalted on C-18 microcolumns following standard procedures. After desalting, digested peptides were diluted in an approximate concentration of 1.5 µg/µL.

After dissolving each sample in 5% acetonitrile, 0.5% formic acid, a total of 1 µg of digested peptides was loaded into a one-dimensional (1D) nano-flow LC-MS/MS system (Eksigent, Germany) equipped with an in-line pre-microfilter (Scivex, USA). Peptides were separated on a monolithic C18 column Chromolith RP-18r (Merck, Germany) of 15 cm length and 0.1 mm internal diameter during a 90 min gradient from 5% to 40% Mobile phase B [90% acetonitrile, 0.1% formic acid], Mobile phase A was 0.1% Formic Acid, with a controlled flow rate of 500 nL per min and 30 min of column regeneration. LC was coupled to MS using an nESI source; MS analysis was performed on an Orbitrap LTQ XL mass spectrometer (Thermo, Germany). Specific tune settings for the MS were set as follows: spray voltage was set to 1.9 kV using a 30 µm inner diameter needle (PicoTip Emitter; NewObjective, USA); temperature of the heated transfer capillary was set to 180 °C. FTMS was operated as follows: fullscan mode, centroid, resolution of 30,000, covering the range 300–2,000 m/z, and Cyclomethicone was used as lock mass. Each full MS scan was followed by seven dependent MS/MS scans, in which the ten most abundant peptide molecular ions were dynamically selected, with a dynamic exclusion window set to 60 s and exclusion list set to 500. Dependent fragmentations were performed in CID mode, with a normalised collision energy of 35, iso width of 1.0, activation Q of 0.250 and activation time of 30 ms. Ions with unassigned or +1 charge were excluded from fragmentation. The minimum signal threshold was set to 500.

## 3 Data analysis

We followed database dependent and independent approaches for quantifying protein abundance. The abundance of the proteins obtained after applying the SEQUEST algorithm [5] was estimated from the peak areas of the three most abundant peptides assigned to each protein employing Proteome Discoverer software (Thermo). A database independent strategy based on the ProtMax software [6] was additionally used to reduce bias related to the poor presence of Pine nuclear proteins in databases. The individual m/z and abundances were determined and quantified using the ion count method following the recommendations previously given by Egelhofer et al. [6].

Data preprocessing steps of both targeted and untargeted strategies were performed following the recommendations given by Valledor and Jorrín [7]. In first place, missing values were imputed employing a sequential K-nearest Neighbour algorithm (KNN), then proteins were filtered by its consistency (being present in all three replicates of one treatment or have 4 values unequal 0 within the 9 analysed samples), and its abundance (this parameter was only considered for MAPA analysis since it is necessary to remove the background noise).

Later, data was normalised following a sample-centric approach (TSS) followed by a log transformation.

To avoid bias caused by considering non-nuclear proteins, abundances of all proteins and m/z were corrected by using the average abundance of the two most stable nuclear proteins present in all samples (Pp003257_2-Pp003276_2 and PITA_000008893-RA, annotated as histones) or the abundance of its corresponding peptides when doing MAPA analysis. The stability of these proteins during the experiment was determined by a pairwise comparison approach considering all detected protein species following the method described by Vandesompele et al. [8]. The results (preprocessed data) of the targeted and untargeted approaches are presented in Appendix A, Appendix A, respectively.

### 3.1 Protein identification

The identification of proteins in our database dependent approach followed a sequential strategy. First, three in-house protein databases were constructed from *​Pinus pinaster* transcriptome v. 3.0 [9], *Pinus taeda* genome v. 1.01 [10], and *Pinus radiata* sequences available in public databases following the procedure described by Romero-Rodríguez et al. [11]. Databases were annotated against UniProtKB/Swiss-Prot and UniProtKB/TrEMBL Viridiplantae using sma3s script [12] and functionally classified through the Mercator pipeline annotation [13] according to MapMan ontology [14]. SEQUEST algorithm included in Proteome Discoverer was used for the identification of proteins employing a 5% false discovery rate (FDR), two peptides per protein, medium confidence, and an XCorr score above 1.8 as identification threshold. Unknown and unannotated proteins were analysed with PlantTFcat tool [15] and blasted against PlnTFDB [16] transcription factors database in order to identify transcription factors and nuclear regulators. Contaminant proteins not belonging to the nuclei or endoplasmic reticulum were dropped from the analysis based on their annotations. Databases are provided as Supplemental material 1; up-to-date versions of these databases will be available at www.valledor.info.

## Footnotes

Appendix A Supplementary data associated with this article can be found in the online version at doi:10.1016/j.dib.2016.03.074.
